# Deciphering Plant Chromatin Regulation via CRISPR/dCas9-Based Epigenome Engineering

**DOI:** 10.3390/epigenomes5030017

**Published:** 2021-08-24

**Authors:** Annick Dubois, François Roudier

**Affiliations:** Laboratoire Reproduction et Développement des Plantes, Université de Lyon, ENS de Lyon, UCB Lyon 1, CNRS, INRAE, 69364 Lyon, France; francois.roudier@ens-lyon.fr

**Keywords:** CRISPR-dCas9, epigenome editing, epigenome engineering, chromatin, plant

## Abstract

CRISPR-based epigenome editing uses dCas9 as a platform to recruit transcription or chromatin regulators at chosen loci. Despite recent and ongoing advances, the full potential of these approaches to studying chromatin functions in vivo remains challenging to exploit. In this review we discuss how recent progress in plants and animals provides new routes to investigate the function of chromatin regulators and address the complexity of associated regulations that are often interconnected. While efficient transcriptional engineering methodologies have been developed and can be used as tools to alter the chromatin state of a locus, examples of direct manipulation of chromatin regulators remain scarce in plants. These reports also reveal pitfalls and limitations of epigenome engineering approaches that are nevertheless informative as they are often associated with locus- and context-dependent features, which include DNA accessibility, initial chromatin and transcriptional state or cellular dynamics. Strategies implemented in different organisms to overcome and even take advantage of these limitations are highlighted, which will further improve our ability to establish the causality and hierarchy of chromatin dynamics on genome regulation.

## 1. Introduction

Chromatin mechanisms play a major role in fine-tuning gene regulation in eukaryotes. The main feature of chromatin is the wrapping of genomic DNA around a protein octamer of four pairs of histones H2A, H2B, H3 and H4, altogether constituting the nucleosome. Nucleosomes prevent DNA from supercoiling and have evolved as a non-specific, passive barrier to limit DNA accessibility [1]. Chromatin can be modified at the DNA level through cytosine methylation, or through post-translational modifications of histones. Histone modifications encompass lysine or arginine methylation, lysine acetylation, lysine ubiquitination or serine phosphorylation, to name a few. In a combinatorial manner, these modifications eventually alter the structure and organisation of chromatin, thereby modulating gene regulatory processes. For example, histone H3K4 di- and tri-methylation as well as acetylation on many residues are usually associated with actively transcribed genes whereas H3K9 and H3K27 di- and tri-methylation are mostly associated with a more compact chromatin state that is restrictive to transcription [2]. Chromatin-based regulation of gene expression is very dynamic and its role in the determination and maintenance of cell identity has been highlighted in all eukaryotes. In plants, global perturbation of chromatin homeostasis at the organism level through knocking-out chromatin regulators often leads to pleiotropic phenotypes that are difficult to interpret, notably because of the intricate relationships between the numerous chromatin regulatory functions. Addressing chromatin functions in vivo is also intrinsically difficult because most molecular connections between chromatin regulation and transcription are correlative, and the causality required to disentangle direct and indirect effects remains a challenge to establish.

The use of clustered regularly interspaced short palindromic repeats (CRISPR) and CRISPR associated (Cas) technology has allowed, within the last few years, a significant leap forward in altering genome function and activity in a controlled and locus-specific manner. Recent transposition of this methodology to epigenome engineering already bears promising fruits in understanding chromatin function in many cellular events. The most commonly used CRISPR type II systems are derived from the adaptative immunity response systems present in the majority of archea species and in about half of bacterial species that use non-coding RNA to guide the Cas9 nuclease to cleave specific viral DNA sequences [3]. Other Cas9 orthologs exist with different DNA contacting and cleavage modalities, such as the Cas9 from *Streptococcus aureus*, or even different systems such as the Cas12a derived from *Francisella novicida* (also named Cpf1) [4], or the type I CRISPR system harnessed from *Clostridium* species [5]. In a CRISPR-mediated genome editing experiment, a single guide RNA (sgRNA) suffices to direct DNA cleavage by Cas9 at a specific genomic location and generate mutations (knock-out) or insertion (knock-in) via DNA repair. Mutations in the catalytic sites D10A and H840A of Cas9 abolishes DNA cleavage while preserving specific recognition of the target sequence. Thus, in a variety of applications including epigenome editing, this catalytically dead Cas9 (dCas9) is used as a synthetic platform to recruit specific proteins or enzymes to a given locus, without affecting its DNA sequence.

CRISPR-dCas9 epigenome engineering can be achieved via two main ways. Transcriptional engineering in which a transacting factor is targeted to a gene regulatory region and destabilizes or triggers a shift in the local chromatin state, and direct epigenome editing in which a chromatin modifier is recruited through dCas9 for locus-specific perturbation of a specific epigenetic regulation. Each of those synthetic approaches was shown to function to some extent in plant and animal systems. These approaches have multiple interests in developmental biology, as they are locus-specific and can be rendered cell-specific or inducible. Furthermore, direct epigenome editing allows to specifically question the causal relationship between chromatin regulator functions and transcriptional regulation dynamics along developmental pathways.

Available CRISPR-dCas9 tools in plants has been reviewed recently [6,7]. In this review, after summarizing in vivo implementations to study gene regulation and function in plants (Supplementary Table S1), we highlight how these methodologies enable assessing the direct function of chromatin regulators as well as more complex interactions including cooperativity, antagonism or neutrality between chromatin regulatory pathways. As most advanced reports are using animal systems, we then discuss how epigenome editing could be implemented in combination with other emerging techniques such as single-cell approaches, to get a deeper understanding of the impact of chromatin-based mechanisms in the regulation of plant development.

## 2. Advances in CRISPR-dCas9 Epigenome Editing in Plants

### 2.1. Transcriptional Engineering in Plants

Efficient transcriptional engineering relies on dCas9 fusion or assembly with transcription activators or repressors, aiming at -so-called- “programmable” gene expression modulation. Transcription modulation is an indirect form of epigenome editing, as induced transcriptional changes can bypass, destabilize or even shift one chromatin state into another. In this case, the intended goal of transcriptional engineering is to override chromatin regulation and it can therefore be used to assess the strength and resilience of the chromatin state over a transcriptional alteration. Select examples of such approaches that successfully allowed targeted transcriptional activation (CRISPRa) or repression (CRISPRi) in plant cells are summarized below.

#### 2.1.1. Locus-Specific Transcriptional Activation (CRISPRa)

**Figure 1:**
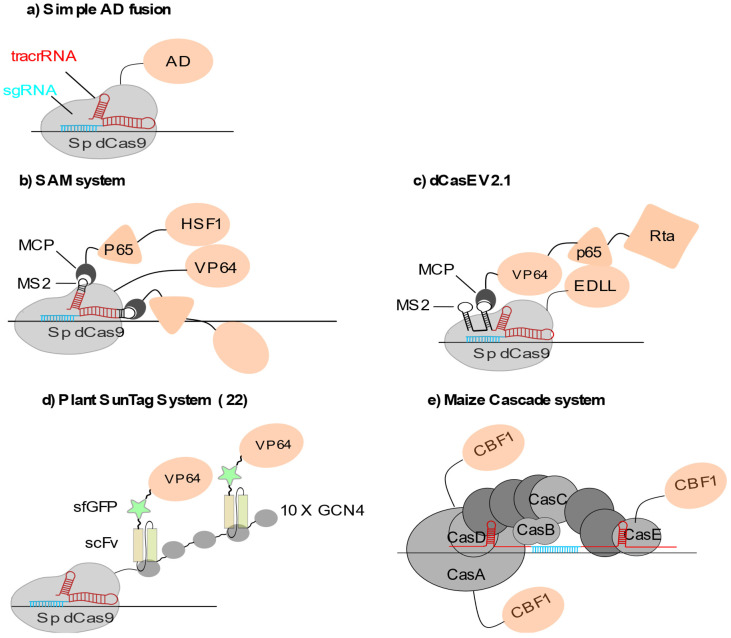
CRISPRa systems used in plants: (**a**) Simple dCas9-Activator domain (AD) fusions. AD can be either VP64, EDLL or the VP64-p65-Rta (VPR) tripartite activator. (**b**) Multiplex AD recruitment through MS2 fusion to sgRNA. (**c**) dCas-EV2.1 combining sgRNA2.1-mediated VPR multiplex recruitment and EDLL domain fusion. Multiplex recruitment is performed through MS2. (**d**) Plant SunTag system, using 10× GCN4 to recruit single chain variable fragment (scFv) antibodies fused to GFP and VP64 in multiplex. (**e**) Cascade system, in which efficient targeted transactivation is reached through multiplexing AP2-family CBF1 activators by fusion to three out of five Cas subunits.

Locus-specific transcriptional activation has been routinely achieved by fusing or recruiting various transcriptional activators to the dCas9. The most widely employed activator in plants so far is the synthetic VP64 domain, which consists in four tandem copies of the *Herpes simplex* viral protein 16 (amino acids 437 to 447). Other potent activators used in plants include the VP64-p65-Rta, or *VPR*, which is a composite of VP64 combined with the activation domains found at the C-terminus of RelA NF-kappaB (p65) and at the C-terminus of the Epstein–Barr virus Rta protein [8], the heat shock factor one (HSF1) trans-activation domain (TAD) [9] or another TAD made of a short motif, termed ‘EDLL’, derived from the plant ERF/EREBP family of transcription factors [10]. In these different approaches, direct dCas9 fusion with VP64 [11], the EDLL peptide [10,12] or the human HSF1 [12] resulted in relatively moderate target gene activation (Figure 1a). Systematic comparison of distinct activator domains using a transient CRISPRa assay in *Nicotiana benthamiana* leaves showed that direct fusions to dCas9 lead to gene activation with roughly the same order of magnitude, no matter which activator is used [8].

In animal cells, multiplexed recruitment of a transcription activator by the dCas9 resulted in significantly stronger gene activation, probably through a more efficient recruitment of the transcriptional machinery, as exemplified for VP64 in HEK293T cells [13,14]. This can be achieved using modified sgRNAs or fusion of multimeric protein adaptors to the dCas9 protein. These sgRNA2.0 can be engineered in different ways to favour the multimerization of transcription modulators. For example, RNA aptamers can be either inserted into the tetraloop and the stem-loop 2 of the sgRNA scaffold sequence (Figure 1b) [15]. They can also be inserted at the 3′ end of the sgRNA (Figure 1c) [16]. These RNA aptamer systems are derived from the RNA bacteriophages MS2 or PP7 systems, where the bacteriophage coat protein is able to specifically bind to an RNA stem-loop structure present in the viral genome [17,18].

The MS2 or PP7 coat proteins are directly fused to the transcription activation or repression domain allowing effector multiplexing at the dCas9 surface (Figure 1b). Such multiplexing strategies have been successfully implemented in plants [11]. Their outcome can be further enhanced by combining direct dCas9 fusion to VP64 with the recruitment of additional activation domains (HSF1 TAD domain and p65) through MS2-modified sgRNAs as described for the synergistic activation mediator (SAM) system [15,19] or the dCas9-TV system [20]. These combinatorial systems have proven their effectiveness on synthetic reporters [8] and on diverse endogenous plant loci involved in biotic or abiotic stress resistance including *WRKY30*, *RLP23* and *CDG1* in *Arabidopsis thaliana*, in development such as *GW7* and *ER1* in rice [20], or in secondary metabolism like *NbDFR* and *NbAN2* in *Nicotiana benthamiana* for instance [8]. Furthermore, RNA-seq analyses performed either on *Arabidopsis* stable lines [20] or *Nicotiana* transient transformations [8] strongly suggest that these approaches are highly specific, with little, if any, off target effect. Similarly, combining direct dCas9-VP64 fusion with the recruitment of MCP-VP64 via sgRNA2.0 was also efficient for targeted gene activation [21]. Another way to recruit multiple VP64 domains is to fuse an array of repeated peptides to the dCas9. The plant version of the SunTag system [22], in which the dCas9 is fused to repeats of a 22 aa GCN4 peptide and co-expressed with an anti-GCN4 antibody chain fused to the transcription modulation domain, showed high efficiency for targeted transcriptional activation of *FWA*, a gene involved in flowering time control in *Arabidopsis* (Figure 1d) [23].

Exploiting the orthologous type I-E CRISPR-Cascade [24], another methodology was recently developed in maize using the C-terminal transcriptional activation domain (TAD) of the *Arabidopsis* AP2-family transcription factor CBF1 fused to three CRISPR-associated complex for antiviral defense (Cascade) proteins [5]. This is a multimeric complex made of three to five proteins depending on the subtype. Upon DNA opening, an R-loop is generated in which the 32 to 33 nt crRNA spacer hybridizes with the target DNA sequence. After target recognition by Cascade, a single-strand DNA exonuclease, Cas3 is recruited to the Cascade complex [25]. This system is likely to be more specific that its Cas9 counterpart as it is based on the formation of a 32nt-long RNA-DNA heteroduplex. Furthermore, the short PAM sequence makes it widely usable in a vast variety of sequence contexts. In terms of engineering, DNA recognition and cleavage are physically separated on different protein subunits, with the described system simply lacking the Cas3 nuclease [5]. Targeted activation of a synthetic reporter gene was greatly enhanced by combining three out of five subunits in the form of CBF1-TAD fusions as compared to the use of single fusion proteins incorporated in the complex (Figure 1e). This demonstrates a cooperative effect between the different activation domains, which however was not observed when the endogenous *R* gene, involved in the regulation of anthocyanin biosynthesis, was targeted [5].

#### 2.1.2. Locus-Specific Transcriptional Repression (CRISPRi)

**Figure 2:**
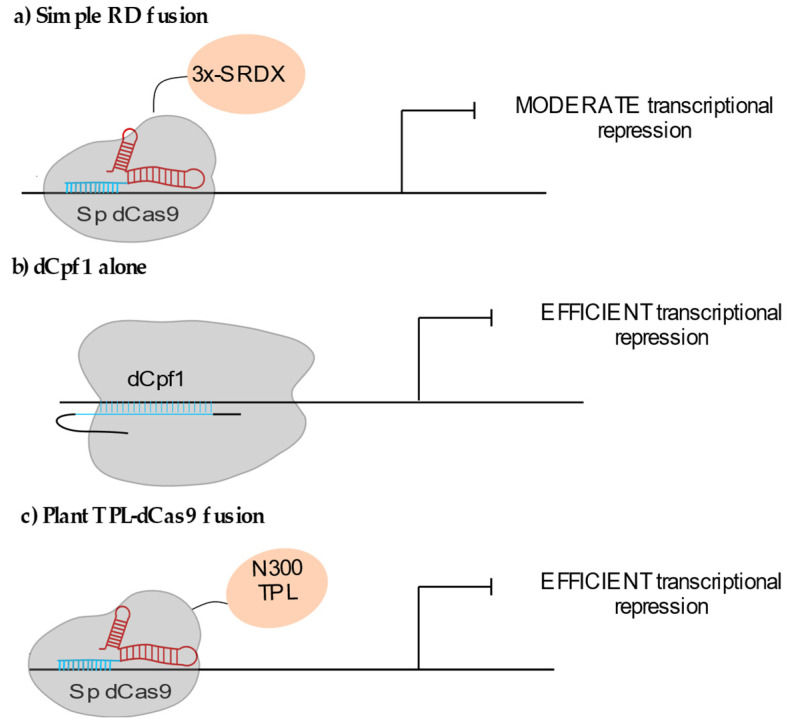
CRISPRi systems used in plants: (**a**) Fusion of the repressive 3x-SDRX domain to dCas9 lead to moderate gene knockdown. (**b**) The dCas9-related nuclease dCpf1 (or dCas12a) alone affects gene expression. (**c**) The 300 AA N-terminal domain of the transcriptional co-repressor TOPLESS (TPL) allows efficient gene repression.

Synthetic transcriptional repressors fused or recruited to the dCas9 have also been used successfully in plants, although to a lesser extent than the CRISPRa strategy. The dCas9 alone was reported as efficiently inhibiting transcription when targeted to the immediate proximity of the transcription start site of the synthetic *NOS* promoter fused to the luciferase in transient assays in *Nicotiana benthamiana* (i.e., <=33 bp from TSS) [26]. The repressor domain mostly used so far is the ERF-associated amphiphilic repression (EAR) SRDX domain. Triple SRDX (3x-SRDX) efficiently repressed transcription when fused to either DNA binding factors [27] or transcription-activator-like effectors [28]. When fused to dCas9, 3x-SRDX only led to moderate knockdown effects on the endogenous *AtCSTF64*, *MiR159a* and *MiR159b* loci (Figure 2a) [11]. As expected, targeting the vicinity of the TSS increased gene repression, whereas multiplexing sgRNA did not significantly improve efficiency [11,26]. The use of orthologous dCas9 systems such as the dCpf1-3x-SRDX fusion also triggered reproducible knockdown (up to 90% reduction in mRNA accumulation) of the endogenous *MIR 159b* gene in *Arabidopsis* [29]. dCpf1 alone was shown to lead to a repression level similar to the one observed using dCpf1-3x-SRDX (Figure 2b) [30]. Parameters such as the different target recognition mechanisms of the two systems or distinct dCas9 or dCpf1 residence time at the targeted locus might also influence the final transcriptional output [31].

Two recent studies report the use of the 300 amino acids N-terminus (N300TPL) of the plant transcriptional co-repressor TOPLESS as another repressive domain for CRISPRi strategies. In both cases local recruitment of N300TPL via dCas9 inhibited target gene transcription (Figure 2c) [32,33]. The TPL N-terminus domain that is able to interact with histone deacetylases [34] and with the core Mediator domain [33], could possibly maintain the transcription initiation complex in a poised state. This domain seems very promising to achieve locus-specific repression in plants.

### 2.2. Epigenome Editing to Manipulate Plant Chromatin Homeostasis

Chromatin homeostasis results from a dynamic equilibrium between multiple pathways that involve a plethora of DNA regulatory factors, histone variants and modifiers as well as nucleosome remodellers. Disentangling these different mechanisms to understand the function of each one in vivo, and its relationship with the others, remains quite challenging. By enabling local recruitment of a given chromatin regulator (CR), CRISPR/dCas9 tools have already started to provide precious insights about the causality of the function of specific epigenetic factors in relation to transcription, in a given genomic and cellular context.

#### 2.2.1. Manipulating Plant DNA Methylation

**Figure 3:**
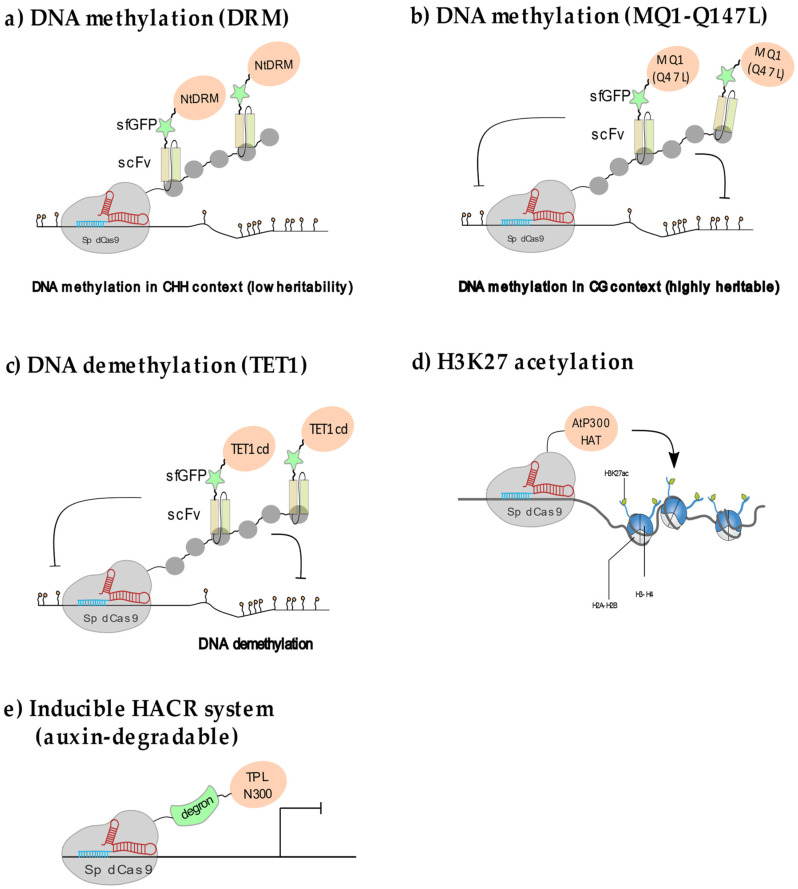
Epigenome editing in plants: (**a**) DNA methylation through multiplex NtDRM catalytic domain recruitment using the SunTag system leads to ectopic methylation preferentially in the CHH context. (**b**) Targeted methylation in the CG context, leading to highly specific and heritable methylation can be obtained using a low DNA affinity variant of the bacterial MQ1 methylase, MQ1(Q147L) (**c**) Locus-specific demethylation is efficiently achieved through SunTag-targeted recruitment of the human TET1 demethylase catalytic domain. (**d**) Local recruitment of the *Arabidopsis* HAT1 through direct fusion to the dCas9 leads to moderate gene activation. (**e**) An auxin-controllable version of dCas9 (HACR system) fused to TPL relieves transcriptional repression in an inducible manner.

In plants, cytosine methylation is found in the CG, CHG and CHH sequence contexts (where H is adenine, cytosine or thymine) and is controlled by the action of de novo and maintenance DNA methyltransferases as well as demethylases. DNA methylation is highly enriched over repeated sequences and present over some gene regulatory regions, where it is associated with transcriptional silencing [35]. In contrast to the situation in mammals, DNA methylation in plants is largely established through prominent RNA-directed DNA methylation (RdDM) pathways (described in [35,36]). In a pioneering effort, DNA methylation was targeted to the *Arabidopsis FWA* locus through multiplex recruitment of the *Nicotiana tabacum* DRM methyltransferase catalytic domain (NtDRMcd) via the SunTag system fused to dCas9 [23] (Figure 3a). Reaching a sufficient level of DNA methylation to achieve stable silencing required to use multiple sgRNAs as landing pads and to maintain the expression of the system during two successive generations. Furthermore, methylation heritability was conditioned upon the levels of CG methylation at the *FWA* locus. The immediate drawback of this approach, also reported for similar designs in animal cells [37], is that the initial NtDRM effector domain fusion with scFv (which recognizes the GCN4 repeat in the Sun Tag) generated high levels of ectopic CHH methylation all along the plant genome. This effect could be attenuated by eliminating one NLS in the scFv-NtDRM protein therefore reducing the amount of methyltransferase into the nucleus [23]. Targeted recruitment of the CG-specific bacterial methyltransferase MQ1 also resulted in the de novo CG methylation of many locations in the genome in addition to the targeted locus [38,39]. This ectopic effect was diminished by using an attenuated MQ1 (Q147L) variant with lower DNA binding affinity in both mammalian and plant cells [38,40] (Figure 3b).

#### 2.2.2. Locus-Specific Demethylation in Plants

Demethylation can occur either passively through DNA replication in the absence of maintenance machinery or actively through enzymatic modules derived from different classes of demethylases such as the mammalian TEN-ELEVEN TRANSLOCATION1 (TET1) protein family that promotes demethylation through a base excision repair pathway [41]. In *Arabidopsis* demethylases include REPRESSOR OF SILENCING 1 (ROS1), DEMETER (DME), DEMETER-LIKE 2 (DML2) and DEMETER-LIKE 3 (DML3). These enzymes directly remove methylated cytosines regardless of the sequence context [35]. The effect of local demethylation on transcript accumulation was assayed using a modified SunTag system in which the dCas9 recruits multiple catalytic domains of the human demethylase TET1cd [42] (Figure 3c). Using this system, the transcription of two types of heavily methylated domains, the *FWA* and the DNA-type transposon *CACTA1* loci, was successfully reactivated without detecting ectopic demethylation events in the locus vicinity or elsewhere in the genome [42]. Whereas stable demethylation of the *FWA* promoter could be maintained after the Sun-Tag TET1 cd construct was segregated out, demethylation was incomplete for the *CACTA1* elements, and re-methylation and re-silencing occurred after removal of the targeted demethylation construct. One promising direction to develop targeted DNA demethylation could be through the use of third generation sgRNAs. In this system, an RNA aptamer that directly inhibits endogenous DNA methyltransferases is grafted onto the sgRNA stem-and tetraloops. The efficiency of this strategy was recently demonstrated in mammalian cells for DNMT1 inhibition using the CRISPR-DIR system [43].

#### 2.2.3. Manipulating Plant Histone Acetylation and Methylation

N-terminal histone lysine acetylation is usually associated with active transcription as it reduces DNA–histone binding strength and favors DNA accessibility by promoting nucleosomes spacing [2]. In animal cells, targeted histone H3K27 acetylation is sufficient to locally enhance transcription at permissive loci (i.e., transcribed loci). A handful of studies performed in cultured human cells show that transcription can be locally enhanced through the targeted recruitment of the human histone acetyltransferase P300, which resulted in efficient, locus-specific H3K27 acetylation on promoters and enhancers in dividing embryonic cells [14] and differentiated cells [44]. Initial attempts at manipulating H3K27 acetylation in plants used the mammalian p300 histone acetyltransferase fused to dCas9. This construct efficiently led to the doubling of H3K27ac levels when targeted to the promoter of the *FLOWERING TIME* (*FT*) locus, a major regulator of flowering time. However, the resulting increase in H3K27ac had little effect on *FT* mRNA levels and slightly affected flowering time [45]. This could be due to limited efficacy of the human co-activator in recruiting the plant transcription machinery. More recently the catalytic domain of the ubiquitous lysine acetyltransferase AtHAT was fused to the dCas9 and targeted to the promoter of the *AREB1* gene (Figure 3d), that is involved in drought stress tolerance in *Arabidopsis* [46]. Although the acetylation level at the locus was not directly measured, the transgenic lines showed a moderate increase in *AREB1* transcripts that was sufficient to increase drought stress tolerance [47]. More experiments are needed to understand fully whether and how perturbing H3K27ac impacts transcription in plants.

Histone methylation is associated with various transcriptional outputs depending on the modified lysine. For example, histone H3K4 and H3K36 di- and tri-methylation are associated with active transcription, whereas H3K9me2 and H3K27me3 are preferentially found over silent repeated sequences or repressed genes, respectively. In plants, numerous histone methyltransferases containing a catalytic SET (Su(var), E(z), and Trithorax) domain exist but only one experiment using a dCas9-SET fusion has been reported so far. The SET domain of KRYPTONITE (KYP), an H3K9 methyltransferase from *Arabidopsis* was targeted to the *FT* locus through a dCas9/MS2 system, with no success at locally editing the histone post translational modification [45]. Still very few studies report dCas9-based epigenome editing approaches in plants and off-target activity analyses are still scarce and limited so far to works involving targeted DNA methylation or demethylation [23,42], with only three studies estimating off-target activity of CRISPRa systems through RNA-seq [8,23,38].

## 3. Epigenome Editing to Interrogate the Effect of Chromatin Regulator Interactions on Transcription

Although experimental data remain scarce in plants and are mostly restricted to proof-of-concept studies, epigenome editing technologies implemented in animals have raised interesting insights that could be directly transposed in plants. Furthermore, in addition to studying the function of individual chromatin factors in regulating transcription, CRISPR/dCas9 also enables to assess the functional relationship between chromatin regulators (CRs) and DNA binding proteins and identify synergies or antagonisms between and among them.

### 3.1. Positive Crosstalks between Chromatin Regulator Pathways

In CRISPRa experiments, co-targeting of histone acetylation and activating transcription factors (TFs) has a higher effect on transcription than targeting only the transcriptional activator [45]. In *Arabidopsis*, recruiting the mammalian p300 histone acetyl transferase to the *FT* locus allowed local doubling of the H3K27ac level, with no significant effect on *FT* transcript accumulation, which suggests that additional activating inputs on the *FT* regulatory sequences are needed to increase transcription [45]. Conversely, targeted recruitment of the TOPLESS co-repressor N-terminal domain, which was shown to recruit the histone deacetylase HDA19 [34], negatively impacts the transcriptional activity of the *AtMED21* promoter, although the resulting acetylation levels were not tested.

In keeping with this synergic mode of action, co-recruitment of dCas9-P300 with the dCas9-VP64 transcription activator at the *OCT4* locus in embryonic HEK293T cells further enhanced gene expression when compared to the effect measured for the single dCas9-VP64 trans-activation [48]. This suggests that histone acetylation synergistically enhances transcriptional activity with the binding of additional activating factors such as TFs.

Similarly, combination of the repressive activities of a CR and a DNA-binding repressor protein can significantly improve CRISPRi efficiency. For instance, local recruitment of the KRAB repressor domain alone only led to short-term repression of the targeted locus in animal cells. In contrast, co-recruitment of the KRAB repressor domain and the DNMT methyltransferase fused to the dCas9 ensured stable silencing in somatic cells [49]. Such a combinatorial approach was implemented as a CRISPRi optimization strategy in a synthetic reporter-based screen in mammalian cells [50]. More than 20 different effector domains were co-recruited in pairs and screened for their ability to repress transcription. Among the selected combinations, dCas9-KRAB fused to the Methyl CpG Binding Protein2 (MeCP2) performed best on four different endogenous target genes. The CRISPRoff system also combines local recruitment of DNMT3, that methylates but also binds methylated DNA, and KRAB domains, respectively fused to the N- and C termini of the dCas9. Here again, combination of both DNA methylation and DNA-binding repressive activities enabled stable transcriptional repression across cell divisions [51].

As in mammals, transcriptionally active genes in plants are usually marked by acetylated histones H3 and H4, monoubiquitinated histone H2B (H2Bub), and di/trimethylated histone H3 at lysine residues such as Lys-4 and Lys-36 (H3K4me2/3, H3K36me2/3) [52]. Interdependence between these marks, their deposition pathways and their respective contribution to transcription remains poorly understood, despite a plethora of correlative studies. The finding that dCas9-targeted recruitment of the E2 ubiquitin-conjugating enzyme (UBE2A) alone had no consequence on transcriptional activation of genes in human embryonic kidney cells suggests that the role of H2B ubiquitylation in activating transcription might be indirect, via the recruitment methyltransferases responsible for H3K4me3 and H3K79me [53]. However, this study did not show actual UBE2a-mediated H2B ubiquitination at targeted locus, which hampers solid conclusions. Interestingly a recent study in *Arabidopsis*, in which there is no H3K79 methylation pathway [54], suggests that H3K4me3 deposition does not rely on a similar trans-histone crosstalk with histone H2B monoubiquitylation, pointing towards an evolutionary divergence in the epigenetic regulation of an otherwise very conserved process [55].

In mammals, the use of CRISPR-dCas9 engineering has been instrumental in determining that deposition of H3K4me3 actively promotes transcriptional activity. Local recruitment of PRDM9, a histone methyltransferase specifically expressed during mammal meiosis, via a direct and inducible dCas9-PRDM9 fusion increased H3K4me3 deposition and upregulated the expression of several genes with different chromatin contexts [53]. Whereas targeted PRDM9 was sufficient to increase H3K4me3 levels, co-editing of H3K4me3 and H3K79me3 through concomitant recruitment of PRDM9 and the H3K79 methyltransferase DOT1L was necessary to maintain transcriptional activation after 20 days of cell culture, through multiple cell division cycles. The combined targeting of both chromatin modifiers demonstrated cooperative enhancement of long-term transcriptional activation [53].

### 3.2. Complex Interactions between Chromatin Regulatory Functions

#### 3.2.1. Modulation of H3K27 Methylation Reveal Cooperative as Well as Antagonistic Interactions

Polycomb repressive complex (PRC2) is a conserved, multimeric chromatin modifier that catalyses H3K27me3 deposition via the SET-domain containing E(Z) subunit. PRC2 activity is involved in maintaining transcriptional repression [56].

In a human cancer cell line, Ezh2-dCas9 was sufficient to generate H3K27 trimethylation at the *Her2* promoter but this was not sufficient for long-term silencing [57]. Only [58] the combined expression of EZH2-dCas9 and dCas9-KRAB was able to establish transcriptional repression in the long-term [59]. Thus, in highly dividing cells, cooperative activity between transcriptional repressor and PRC2 maintenance appears necessary to establish long-term repression.

In *Medaka* embryos, dCas9-OlEZH2 ubiquitous expression was sufficient for site-specific accumulation of H3K27me3 and long-term target gene repression [58]. Interestingly, DNA methylation impaired targeted H3K27me3 deposition, although dCas9-OlEZH2 local recruitment was detected by ChIP even at highly DNA-methylated loci. This indicates that the antagonist action of cytosine methylation on H3K27me3 deposition cannot be solely explained by the inability of dCas9 to access DNA methylated regions [58]. Anticorrelation between DNA methylation and H3K27me3 accumulation has been described in vitro [60] as well as in vivo in both animal cells [61,62] and plants [63,64]. A recent report further confirms this anticorrelation, in which SssI-mediated CG-methylation targeted to the *FWA* locus triggers ectopic CG methylation at numerous loci over gene bodies. Ectopic CG methylation was shown to reduce the accumulation of H3K27me3 as well as H2A.Z in gene bodies, which supports that CG methylation in plants affects H3K27me3 and H2A.Z accumulation [39].

PRC2 activity can be locally inhibited. A genetically encoded PRC2 inhibitor named EED-binder was used to compete with EZH2 activity in vivo, likely through the titration of EED, another core subunit of PRC2 [65]. When targeted to different loci regulated by PRC2 in induced pluripotent stem cells, inhibition of PRC2 via the EED-binder–dCas9 fusion led to their upregulation and an increase in DNA accessibility as demonstrated by ATAC-seq analysis [66]. These targeted approaches aimed at locally modulating H3K27 methylation could also be useful to assess whether the impact of H3K27-methylated regions can act from a distance, forming loops with transcriptional repression activity [67].

#### 3.2.2. Antagonism between DNA Methylation and Transcription Factors

As described above, efficient silencing of the *FWA* locus in *Arabidopsis* could be obtained through targeted DNA methylation [23], which showed that cytosine methylation at regulatory regions is sufficient to block transcription, possibly by impairing transcription factor binding. In line with this, targeted methylation in animal cells revealed that DNA methylation impacts local interactions with TFs, sometimes resulting in contrasting outputs. During motor neuron differentiation, de novo DNA methylation plays a direct role in regulating the expression of a cascade of transcription factors like ARX and PAX6 that controls the initiation of the differentiation sequence [68]. Knocking out the major DNA methyltransferase DNMT3A resulted in opposite transcriptional changes at the *Arx* and *Pax6* promoters, possibly because the absence of DNA methylation favoured the action of a repressor TF at *Arx* and of an activator TF at *Pax6*. Partial rescue of motoneuron identity was obtained by simultaneously targeting dCas9-DNMT3A fusion both at *PAX6* and *ARX* regulatory regions [68].

This study highlights the huge interest in locus-specific manipulation of DNA methylation to establish direct relations between chromatin regulation and transcription, and also illustrates the intrinsic complexity of predicting how targeted modulation of DNA methylation dynamics might affect the expression of a gene. In line with this, in vitro assays in plants showed that the binding of over three quarters of *Arabidopsis* transcription factors (248 TFs out of 327 tested) is sensitive to DNA methylation, which either inhibits (72%) or favours (4.3%) its binding to a DNA motif [69].

Methylation-sensitive differential TF binding is not the sole mechanism which can explain contrasting transcriptional outputs of DNA methylation. Two studies in *Arabidopsis* reveal how DNA methylation can modulate transcription in opposite ways. A recent screen for methyl readers identified readers with positive [70] as well as negative effects on transcription [71]. Thus, local chromatin context, through recruitment of different readers, can explain contrasting outputs of transcription. CRISPR-dCas9 driven modulation of chromatin status could therefore become a potent way to analyse this local chromatin context and fine-tune or even rewire transcriptional networks at the locus level.

### 3.3. Transcription Factors and Histone Acetyltransferases Have Distinct Effects on the Timing of Transcription

Simultaneous manipulation of histone H3K27 acetylation on the *OCT4* promoter and enhancer regions using a dCas9–SunTag–p300core fusion in cultured fibroblasts showed that targeted recruitment of P300 to both regulatory regions is sufficient to trigger sustained expression, with maintenance of transcriptional activation after 10 days in culture [44]. The same result was reached after only 5 days of expression of the dCas9–SunTag–VP64 synthetic activator. Besides confirming that distinct mechanisms or succession of events are at play, these results also provide an interesting way to control the timing of gene activation and test its impact in a differentiation context.

In keeping with this notion of transcription timing, CRISPR-mediated acetylation via dCas9–p300 was used to study the link between periodic histone acetylation and transcriptional bursting during the circadian rhythm. Heterochronic acetylation of histones was sufficient to alter the frequency of gene expression revealing a direct correlation between acetylation levels at regulatory regions and rhythmic transcriptional burst [72]. Similarly, modulation of histone acetylation using dCas9–P300 and dCas9–HDA8 showed that histone acetylation controls transcriptional burst dynamics during neurogenesis [73].

## 4. Exploiting Current Limitations in Using CRISPR/dCas9 Approaches and Going beyond

The advent of dCas9 technologies has started to provide efficient tools to disentangle complex and interrelated chromatin functions in different organisms. These approaches also revealed some apparent limitations, the exploitation of which could bring deeper insights into chromatin dynamics in vivo and calls for the design of next generation CRISPR/dCas9-based tools.

### 4.1. Locus Accessibility and Chromatin Status Impacts on CRISPR/dCas9 Output

Locus accessibility is an important parameter when conceiving transcriptional or epigenome engineering experiments. Indeed, nucleosomes are a direct physical barrier for Cas9 target binding, both in vitro [74] and in vivo [75]. Furthermore, many reports highlighted the fact that varying DNA accessibility due to a more-or-less permissive chromatin context at targeted loci strongly affects the outcome of a dCas9-based strategy. For instance in mammalian cells, CpG islands hypermethylation in target gene regulatory regions can hamper dCas9 binding, as shown by the inability to retrieve these regions through dCas9 chromatin immunoprecipitation, whereas global DNA demethylation via a treatment with 5′-Azacytidine lifted this barrier [53]. Interestingly, targeting of the same chromatin modifiers via zinc-finger-driven (ZF) fusion proteins in the same conditions worked irrespectively of the DNA methylation status of the loci. The different steric hindrance of dCas9 and ZF, or the fact that ZF contacts DNA in a different manner than dCas9 may account for this difference. This limitation was also observed for mutagenesis screens using active Cas9, in which the chromatin status greatly influences target accessibility [76,77]. Similarly closed chromatin that is targeted by PRC2 and enriched in H3K27me3 is hardly accessible to the active Cas9 and this block can be unlocked using PRC2-inhibitors or local VP64 recruitment to modify local chromatin context [78]. Whether this limitation could be turned into an advantage by creating a dCas9-based sensor of DNA accessibility in vivo will require further investigations. This is apparently at odds with the report in *Medaka*, where dCas9-OlEZH2 which was recruited to target loci with methylated cytosines. However, in this study, cautious design of sgRNAs was performed to target only DNAse I hypersensitive sites obtained from DNAse I-seq data from *Medaka* blastula [58]. This allowed to target a methylated region and unmask DNA methylation vs. PRC2 antagonism. This underlines the importance of prior validation of sgRNA as well as careful a posteriori assessment of dCas9 binding.

Interrogating the epigenome through local perturbation of chromatin dynamics via dCas9–CR fusions can result in variable outputs depending on the target locus chromatin context. In a trivial way, opening up the accessibility of a locus via targeted histone acetylation, for instance, can promote equally the binding of a transcriptional activator or repressor at different loci, hence leading to a different transcriptional response [11,12]. Similarly, the level of transcriptional activation achieved by VP64 multiplex recruitment through the SunTag system can be extremely variable depending on the DNA methylation status of the targeted region [23]. In the case of heavily methylated regions such as the *FWA* promoter, only moderate transcriptional activation could be detected, whereas targeting the non-methylated promoters of *APETALA3* or *CLV3* led to a strong increase in mRNA accumulation. It is not entirely clear whether these differences resulted from a differential access of the dCas9 fusion protein or to a competition between the transactivating effect of VP64 and the silencing action linked with DNA methylation.

### 4.2. Cell-Type Specific Responses to Targeted Chromatin Modifiers

The outcome of targeted epigenome manipulations can also vary depending on cell identity, i.e., on the transcriptional and chromatin status of a given locus at a given developmental time point. Targeting dCas9-VP64 or -VPR to the *Sox1* gene regulatory regions in mice neural progenitor cells led to a heterogeneous response with many transfected cells escaping CRISPRa [79]. This variable response was correlated with distinct cytosine methylation patterns around the *Sox1* TSS and a more homogeneous response was achieved by combining the simultaneous recruitment of the histone acetyltransferase p300 and the DNA demethylase TET1. Plant studies using CRISPR-mediated epigenome engineering so far have been conducted at the whole organism level. Thus, the observed response is likely an average of many different scenarios that can blur interpretations. As single-cell approaches are picking up in plants, especially scRNA-seq [80], transcriptional cell-to-cell variability during development and in response exogenous stimulation is becoming more and more apparent [81,82]. Therefore, it is expected that the action of dCas9–CR fusions will also give heterogeneous outcomes that will have to be carefully monitored.

### 4.3. Combining Epigenome Editing with Cell-Specific Approaches

One complementary leverage in exploiting epigenome editing technologies in plants is the use of inducible or cell-specific control of the CRISPR–dCas9 system. Cas9 expression can be rendered inducible and cell-specific [83,84] and, although still scarce, similar approaches can be used with dCas9 platform.

Conditioning of dCas9 activity to auxin concentration was achieved by fusing the dCas9–C-terminus to an auxin dependent degron (31). In this HACR system (Figure 3e), the dCas9–degron was used to recruit the TOPLESS repressor domain TPL300 to a minimal promoter that was able to maintain transgene expression at relatively low levels in root cells. When a pulse of exogenous auxin was applied, the repressive effect was quickly relieved resulting in rapid and transient transgene activation within 1 h [32]. Expressing the dCas9-TPL300 repressor in the plant vasculature to target the regulatory regions of the *AtMED21* gene led to cellular identity reprogramming, consistent with reduced target gene expression. Further work demonstrated that TPL300-mediated gene co-repression involves members of the mediator complex [33]. Accurate manipulations of CR activities at select loci and in specific cell types and developmental time points will undoubtedly become essential tools, not only to question the function of endogenous genes via programmable transcriptional control but also to understand the role of chromatin remodelling in development.

## 5. Conclusions

This report summarizes current approaches to locally modify plant chromatin homeostasis through the use of dCas9 and address the function of individual chromatin regulator activity. While in vivo studies based on dCas9-based approaches are slowly cranking up in plants, they should be more generally accompanied by transcriptomic and epigenomic analyses not only to verify target specificity but also to assess efficiency and consequences in distinct cellular and molecular contexts. Considering all proof-of-concept works in plants (see Supplementary Table S1), so far very few studies have compared different tools on the same platforms, which renders quantitative comparisons particularly difficult. In this respect, screens based on transient assays such as the one described in [8] would be very informative and help reaching higher ease and efficiency of use. CRIPSR–dCas9 approaches and related systems are gradually adding to the panoply of methodologies available to assess the causal function of chromatin regulators and the interactions between epigenetic pathways. Combining CRISPR–dCAs9 approaches with single-cell techniques appears particularly promising to build a thorough understanding about the role of chromatin-based mechanisms in regulating plant growth and development.

## Data Availability

The data that support the findings of this study are available from the corresponding author upon reasonable request.

## Supplementary Materials

The following are available online at https://www.mdpi.com/article/10.3390/epigenomes5030017/s1, Table S1: Summary table of epigenome engineering studies in plants.
